# Chiral Nematic Cellulose Nanocrystal Films for Enhanced Charge Separation and Quantum-Confined Stark Effect

**DOI:** 10.1021/acsnano.4c04727

**Published:** 2024-10-09

**Authors:** Gur Aminadav, Omer Shoseyov, Shylee Belsey, Daniel Voignac, Shira Yochelis, Yael Levi-Kalisman, Binghai Yan, Oded Shoseyov, Yossi Paltiel

**Affiliations:** † Department of Applied Physics, The Hebrew University of Jerusalem, Jerusalem 9190401, Israel; ‡ Department of Plant Sciences and Genetics in Agriculture, Robert H. Smith Faculty of Agriculture, Food and Environment, The Hebrew University of Jerusalem, Rehovot 7612001, Israel; § The Center for Nanoscience and Nanotechnology, The Hebrew University of Jerusalem, Jerusalem 9190401, Israel; ∥ Department of Condensed Matter Physics, Weizmann Institute of Science, Rehovot 7610001, Israel

**Keywords:** CISS effect, chirality, CNC, charge separation, stark effect, photovoltaic cell

## Abstract

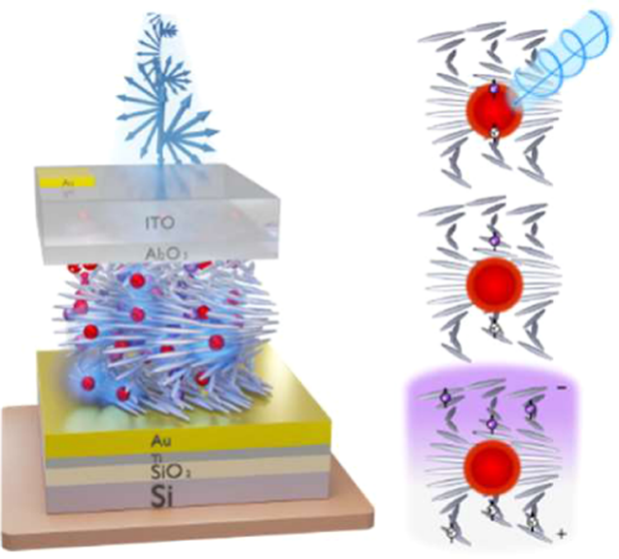

Efficient charge separation is essential in various optoelectronic systems, yet it continues to pose substantial challenges. Building upon the recent evidence that chiral biomolecules can function as electron spin filters, this study aims to extend the application of chirality-driven charge separation from the molecular level to the mesoscale and supramolecular scale. Utilizing cellulose nanocrystals (CNCs) derived from cellulose, the most abundant biomaterial on Earth, this research leverages their self-assembly into chiral nematic structures and their dielectric properties. A device is introduced featuring a chiral nematic hybrid film composed of CNCs and quantum dots (QDs), decorated with iron oxide nanoparticles. Using the quantum-confined Stark effect (QCSE) to probe charge separation, we reveal significant sensitivity to the circular polarization of light and the chiral nematic structure of the film. This approach achieves effective, long-lasting charge separation, both locally and across length scales exceeding 1 μm, enabling potential applications such as self-assembled devices that combine photovoltaic cells with electric capacitance as well as optical electric-field hybrid biosensors.

## 1 Introduction

The efficient separation of photogenerated charge carriers is a fundamental cornerstone in a variety of optoelectronic systems, particularly in photovoltaic applications such as artificial photosynthesis, photoelectrochemical water splitting, solar fuel production, and photodetection.^1−3^ In addressing this challenge, the integration of chiral biopolymers^4^ and nanostructures provides opportunities not found in conventional semiconductor devices.^5^ Recent studies show that chiral molecules can act as spin filters,^6−8^ a phenomenon known as the chiral-induced spin selectivity (CISS) effect.^9^ While the precise mechanisms combine several effects, the CISS main mechanism is attributed to spin–orbit coupling that aligns a charge carrier’s spin with its linear momentum as it passes through chiral molecules.^10−13^ This break of symmetry, which is influenced by charge sign, current direction, and the handedness of the molecule, results in differential spin transfer probabilities, favoring the traversal of charges with a specific spin through the chiral molecule and can thus be leveraged for charge separation.

Building on this understanding, Peer et al. employed quantum dots (QDs) and chiral L-α helix polyalanine monolayers to develop a device that achieves efficient charge separation at sub–5 nm length scales without the need for doping.^14^ A related effect was observed in chiral diodes emitting circularly polarized light (CPL).^15^ Furthermore, CISS properties have recently been demonstrated also in polymers,^16^ inorganic materials, and organometallic supramolecular assemblies.^17,18^ Our study investigates the application of chirality-driven charge separation, extending it from the molecular scale to the mesoscale and supramolecular scale by employing cellulose nanocrystals (CNCs) and their inherent ability to self-assemble into chiral nematic films with a helicoidal structure.^19−21^ This extension holds the potential for achieving higher voltages, a prerequisite for practical applications, through the use of a sustainable and low-cost material.

CNCs, produced through controlled acid hydrolysis of cellulose—the most abundant biomaterial on Earth, are sourced predominantly from plants, as well as bacteria, algae, and tunicates. The crystalline rod-like CNCs, composed of d-glucose units, are typically 100–250 nm long and 5–20 nm wide and have a right-handed longitudinal twist. Aqueous suspensions of CNCs, above a critical concentration, spontaneously self-organize into chiral nematic, also known as cholesteric, liquid crystals.^19−21^ In this phase, CNCs collectively align in a distinct pattern: each nanocrystal achieves long-range orientation correlation with its neighbors, and they additionally exhibit a periodic spatial rotation along one axis. This results in a left-handed helicoidal structure, visualized as a stack of layers, each containing uniformly aligned CNCs, and is slightly rotated relative to the layer below. Upon solvent evaporation, this left-handed helicoidal structure can be retained in solid-state CNC films formed through self-assembly.^19−21^ Several mechanisms were proposed to explain the chirality transfer from individual CNC particles to their collective chiral nematic behavior.^21^ These include chiral steric interactions due to the twisted shape of CNC particles,^22−25^ chiral electrostatic interactions stemming from helical charge distribution variation in individual CNCs,^20^ chiral dispersion forces,^26,27^ and electron spin-exchange interactions between chiral CNC particles.^28^ Moreover, prior research indicates that such spin-dependent exchange interactions lead to strong short-range attractive forces between chiral molecules of the same handedness,^29^ like right-handed CNC particles, particularly when charge redistribution and metastable spin polarization in individual chiral molecules occur as these chiral molecules approach each other.^30^ To illustrate spin-dependent exchange interactions, Al-Bustami et al. demonstrated that adsorbing CNC on a Hall probe and drying it induces local magnetization in the substrate.^28^

In light of these strong, albeit not covalent, short-range forces between CNC particles, this study examines whether the CISS effect, previously demonstrated to facilitate charge separation in chiral molecules, can be similarly applied to the chiral nematic order of CNC films. Key to this examination are three pivotal components: first, the ability to control the chiral nematic order of CNC films; second, the ability to control the spin states of photoexcited charges; and third, the ability to probe local charge separations within the CNC film.

The chiral nematic order of CNC films is sensitive to the CNC suspension properties, including pH and ionic strength, as well as to the conditions during solvent evaporation, such as temperature, humidity,^19−21,31−34^ and the application of external magnetic fields or magnetic substrates.^28,35−37^ For discerning the impact of the chiral nematic structure on charge separation, methods that could significantly alter the film’s electrolytic properties, like adjustments in pH, salts, or ionic content, were avoided in our study.^38−40^ Instead, controlled magnetic and temperature modulations were utilized. The influence of magnetism on the chiral nematic order stems from the anisotropy of CNCs’ diamagnetic susceptibility and the impact on electron spin-exchange interactions of the CNCs during film formation.^28^ Additionally, the impact of magnetic fields on the chiral nematic order is significantly enhanced when CNCs are decorated with magnetic nanoparticles.^41,42^ Applying these principles, we developed a device featuring a hybrid film composed of CNCs decorated with iron oxide nanoparticles (IONPs), within which CdSe/ZnS QDs were dispersed. Different film variants were prepared, each subjected to distinct drying conditions involving both magnets and temperature variations that either preserved or disrupted the chiral nematic order.

The control over the spin states of photoexcited charges constitutes the second key aspect of this study. Upon CPL excitation above the bandgap energy, QDs in our device generate electron–hole pairs with specific spin states.^43,44^ The circular polarization of the incident light (right or left) determines whether the electrons are spin-up or spin-down. Past research indicates a rapid spin-flip process for holes in QDs at room temperature, contrasting with the notably slower relaxation of electron spins, which occurs on the nanosecond time scale. This disparity allows for the formation of electron–hole pairs with the equal sign of spin states (*S*_e_,*S*_h_)_±1/2,±3/2_.^45−48^ An extension of the CISS effect—previously observed in chiral molecules—to chiral nematic structures suggests that circularly polarized light, right CPL (RCPL), or left CPL (LCPL) would generate electron and hole pairs with spin states that either favor or impede transfer through the chiral potential of the left-handed chiral nematic CNC film.^9^ Furthermore, the interaction of CNCs with QDs, facilitated by spin–orbit coupling at the surfaces of the QDs, where charge movement is potentially mediated by the abundant OH groups on the CNCs surfaces, can amplify this effect by imprinting CNC’s chirality onto the QDs, as previously observed with chiral ligands.^49−52^ After CPL excitation, electrons with favored spin states are expected to migrate through the CNC film, moving away from the QDs. Conversely, holes, retaining the same spin sign as the electrons, are likely to migrate in the opposite direction. This directional divergence should reduce the likelihood of recombination, provided the spin states of the charge carriers remain unchanged.

To probe the spin-dependent electron transfer mediated by the chiral nematic structure of CNC and the resulting charge separation, we apply photoluminescence measurements, specifically utilizing the quantum-confined Stark effect (QCSE).^53,54^ Typically, under an externally applied electric field, the QCSE manifests as a (red-)shift in the position of the emission peak, accompanied by a decrease in the fluorescence energy emitted.^55−57^ In our setting, we anticipate a durable accumulation of separated, trapped charges, leading to the establishment of local electric fields around the QDs, which manifest as uniformly oriented local dipoles throughout the film. This uniform orientation is predicted by the CISS effect, where the left-handedness of the chiral nematic CNC dictates the direction of efficient transmission for electrons or holes through the film, conditional on their spin state, charge sign, and direction of motion.^9^ The formation of these consistently aligned dipoles around the QDs is expected to trigger the QCSE. Their interaction with any externally applied field could either amplify or attenuate the QCSE, depending on how the external field’s orientation aligns with that of the local dipoles.

A sequence of RCPL and LCPL excitations, along with voltage biases, was applied to devices with varying degrees of chiral nematic order in the CNC-IONPs-QDs films. A significant divergence in spectral shifts between LCPL and RCPL excitations occurred only in devices with a more chiral nematic order. Furthermore, in these chiral nematic devices, the direction of the applied voltage bias affected the magnitude of the spectral shift for a given light polarization excitation. These observations align with the asymmetry expected from a CISS-driven charge separation. Notably, each subsequent light excitation prompted a further spectral shift, indicating the accumulation of local charge separations within the device, even in the absence of an external voltage bias. These local dipoles contributed to significant and enduring charge separation across length scales exceeding 1 μm, manifested as a 7 nm spectral shift using simple symmetric spherical QDs. To reset the device for subsequent measurements, a discharge process was crucial to neutralizing this accumulated dipole.

Our research and device leverage the distinct combination of the chiral nematic structure and dielectric properties of CNC to achieve the effective and long-lived separation of photoexcited charges. Since CNC-based materials are biocompatible, biodegradable, scalable, and exhibit a wide range of mechanical and rheological properties,^19,41,58−63^ this study also promotes the development of sustainable and low-cost CNC-based electronic and optoelectronic applications with useful features.

## 2 Results and Discussion

### 2.1 Device Fabrication

**Figure 1:**
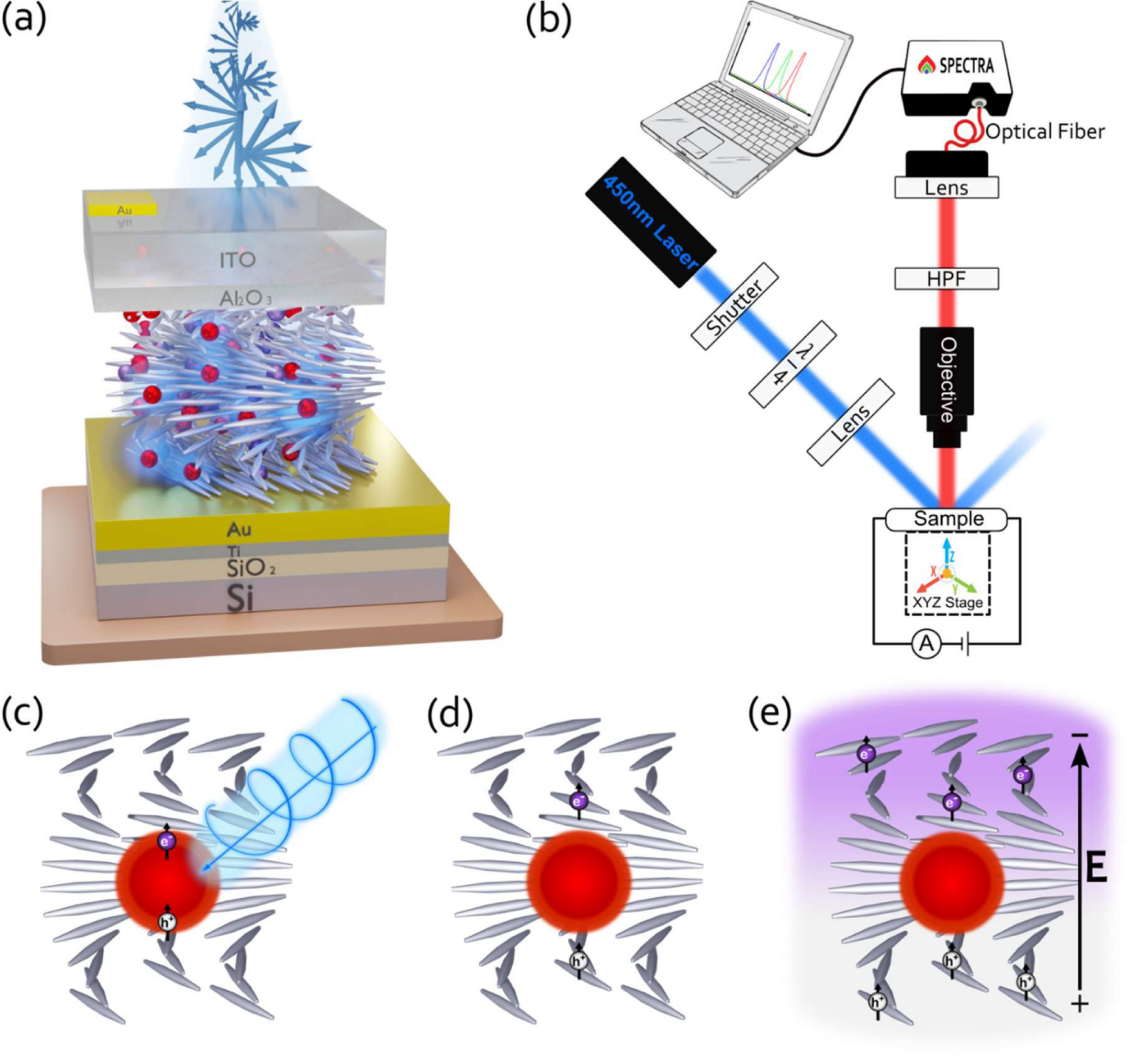
Schematic illustration of the device structure, measurement setup, and charge separation mechanism. (a) Illustration of the device structure. On a Si/SiO_2_ substrate, a thin Ti adhesion layer was evaporated, followed by an Au layer, next a multilayer structure of dispersed core/shell CdSe/ZnS QDs within a CNC-IONPs suspension was deposited and dried, followed by a thin Al_2_O_3_ capping layer. On top of that, a transparent yet conducting layer of ITO was deposited, and finally, a small Au strip was evaporated on top to aid with the bonding procedure. (b) Measurement setup. The sample is connected to a voltage source and positioned on an XYZ stage. The beam is controlled by a shutter to regulate the exposure time of the sample. It then traverses a quarter waveplate to generate circular polarization before being focused onto the sample through a convex lens. The emitted fluorescence is collected by a microscope objective, passes through a high-pass filter, and is then focused using another convex lens. The focused fluorescence is coupled to an optical fiber, which is further connected to a spectrometer, which is in turn connected to a computer for analysis. Charge separation mechanism. (c) A single QD is excited by a circularly polarized laser beam. This excitation follows the optical selection rules and leads to the formation of an electron–hole pair with a specific spin state. (d) The charges, i.e., the electron and hole, undergo tunneling to the chiral environment through the CISS effect. This tunneling mostly occurs along the direction of the chiral axis of the system. (e) Once the charges reach the chiral environment, they become trapped in the trapping sites of the system, creating an electric field gradient along the chiral axis over time. Perpendicular magnetic fields were applied only during drying of the CNC-IONPs-QDs suspension in protocols “S” and “N” to influence the films’ chiral nematic structure, and 3 samples were prepared for each of the 4 drying protocols, resulting in a total of 12 samples. No external magnetic fields were used during the optical or electrical measurements of any film.

The design of the device and experimental setup are illustrated in Figure 1. The experimental setup is designed to investigate charge separation upon photoexcitation of QDs within the CNC-IONPs-QDs film by measuring their emission spectrum both with and without an externally applied electric field. At the core of this device is a multilayer structure, as shown in Figure 1a. The CNC utilized in this study was derived from wood pulp cellulose and processed through sulfuric acid hydrolysis. The majority of CNC particles were 100–200 nm in length and 5–10 nm in width, as characterized by transmission electron microscopy (TEM) analysis (Figure S1). To amplify CNC magnetic susceptibility, a suspension of CNC-IONPs composites was synthesized through the *in situ* growth of IONPs onto CNCs (Figure S2a,b).^41,42^ The resulting IONPs have an average diameter of 7.8 ± 2 nm, as determined by scanning transmission electron microscopy (STEM) (Figure S2c–f). Energy-dispersive X-ray spectroscopy (EDS) analysis confirms the presence of iron oxide on the CNC particles (Figure S2d). X-ray powder diffraction (XRD) measurements (Figure S2g) of the CNC-IONPs sample reveal primary diffraction peaks at 14.7, 16.3, and 22.8°, corresponding to the Miller indices (110), (11̅0), and (200) respectively, as expected for typical CNC materials.^64^ Additionally, a small diffraction peak at 35.7° corresponds to the (311) plane of the iron oxide crystal lattice,^65^ further confirming the presence of iron oxide nanoparticles.^41,42^

Spherical core/shell CdSe/ZnS QDs, with an average diameter of 9.5 ± 0.5 nm (Figure S3), were then dispersed into the CNC-IONPs suspension, leveraging CNC’s effective nanoparticle dispersing capabilities (Figure S4a).^61,62^ As a result, the final hybrid suspension achieved a CNC concentration of 1 wt % and a ζ-potential of −64 ± 3.9 mV (compared to −37 ± 1.6 mV for pure CNC), indicating colloidal stability.^66^ This is a prerequisite for the chiral nematic phase of CNC in suspension.^21^ The CNC-IONPs-QDs suspension was subsequently drop-cast onto a SiO_2_/Ti/Au substrate. Four CNC-IONPs-QDs film variants were produced by applying controlled drying conditions that either facilitated or disrupted the chiral nematic order, as detailed in Section 2.2.

EDS (in SEM) analysis of a dried CNC-IONPs-QDs film, sampling 24 points along a 1.7 μm line running diagonally to the film’s cross-section (Figure S4b,c), reveals the presence of iron, sulfur, cadmium, and zinc and shows that their percent weights are stable across the sampled points. This confirms the presence of both iron oxide nanoparticles and CdSe/ZnS QDs and suggests a well-distributed dispersion of the nanostructures within the dried film.

Dielectric permittivity measurements, conducted using a parallel plate capacitor setup,^67^ revealed the average dielectric permittivity (ε_r_) of the CNC-IONPs-QDs films to be 8 ± 2, surpassing that of pure CNC films, which exhibited an average ε_r_ of 6 ± 2, aligning with prior assessments of pure CNC.^63^

The fabrication process continued postdrying (Figure 1a). A 30 nm Al_2_O_3_ insulation layer was deposited over the dried CNC-IONPs-QDs films. Following this, a 100 nm transparent layer of indium tin oxide (ITO) was sputter-coated on top of the isolation layer. The final step involved the deposition of a narrow Cr/Au electrode on the ITO layer. The active area of the completed device measured 3 mm × 3 mm.

Complementing the fabricated device, a custom measurement setup was designed, as depicted in Figure 1b. This configuration enables the photoexcitation of the QDs by using either LCPL or RCPL. The emitted fluorescence from the QDs is subsequently captured and analyzed by a spectrometer. To add a voltage gradient, a synchronized modulated voltage source connected via the Au electrodes was used, generating an electric field perpendicular to the CNC-IONPs-QDs film surface.

### 2.2 Formation of Chiral Nematic CNC-IONPs-QDs Films

During solvent evaporation of the CNC-IONPs-QDs suspension, the timing of the transition from a flowing suspension of freely moving CNC-IONPs-QDs particles to a “locked” gel-like state with limited mobility is crucial. For the resulting film to exhibit a chiral nematic structure, this transition must take place after CNCs have self-assembled into a chiral nematic phase within the suspension; otherwise, with the premature transition, the film will lack chiral nematic order.^21^ Thus, the drying rate, adjusted via temperature and humidity, is key to the film’s chiral nematic structure.

Furthermore, applying a magnetic field during the drying process was shown to influence the chiral nematic order, a result of the anisotropy of CNCs’ diamagnetic susceptibility and the impact on electron spin-exchange interactions between individual CNCs.^28,29^ During solvent evaporation, charge redistribution occurs across individual chiral CNC particles as they approach each other, leading to spin-dependent electron transport and metastable spin polarization across individual CNC particles due to the CISS effect. This should result in strong short-range spin-exchange interactions between CNC particles.^29^ When CNC is dried on a magnetic surface, short-range magnetic exchange interactions between chiral CNC particles and the perpendicularly magnetized surface become spin-sensitive.^68^ The right-handed chirality of CNC particles means that the magnetization direction affects these interactions (stabilized or destabilized). If the drying is slow enough, these interactions will propagate through the forming CNC structure and are expected to influence the chiral nematic order of the resulting film.^28^

Based on the influence of drying dynamics and magnetic fields, four distinct drying protocols were applied to the CNC-IONPs-QDs suspension. The protocols were chosen for their expected different effects on the formation of the chiral nematic structure. The first device, “O”, was formed through slow drying at 45% relative humidity (RH) and 15 °C for 4 days, which is expected to provide CNCs sufficient time to self-assemble into a chiral nematic order. The second device, “S”, involved the same slow drying conditions (RH = 45%, *T* = 15 °C, 4 days) but on a magnet’s south pole, shown to support the chiral nematic order through its influence on spin-exchange interactions between chiral CNC particles.^28,29^ The magnet also promotes perpendicular alignment of CNCs to the magnetic field, enhancing helicoidal uniformity.^69−71^ Conversely, the other protocols were found to hinder the proper self-assembly of the chiral nematic structure. The third device, “FD”, involved rapid drying at 80 °C for 4 h in an oven, which is anticipated to trigger a premature “locked” state of CNCs, thus preventing the formation of the chiral nematic phase. The fourth device, “N”, entailed slow drying (RH = 45%, *T* = 15 °C, 4 days) on a magnet’s north pole, which was observed to disrupt the chiral nematic order through its opposite influence on spin-exchange interactions between chiral CNC particles,^28,29^ and could even unwind the helicoidal structure, resulting in a more unidirectional morphology.^19−21,31−34,36,42^

The magnetic setups utilized a 200 mT constant field generated by a disk magnet positioned beneath the Petri dish containing the drying sample. After being dried, each CNC-IONPs-QDs film reached a thickness of approximately 2 μm.

**Figure 2:**
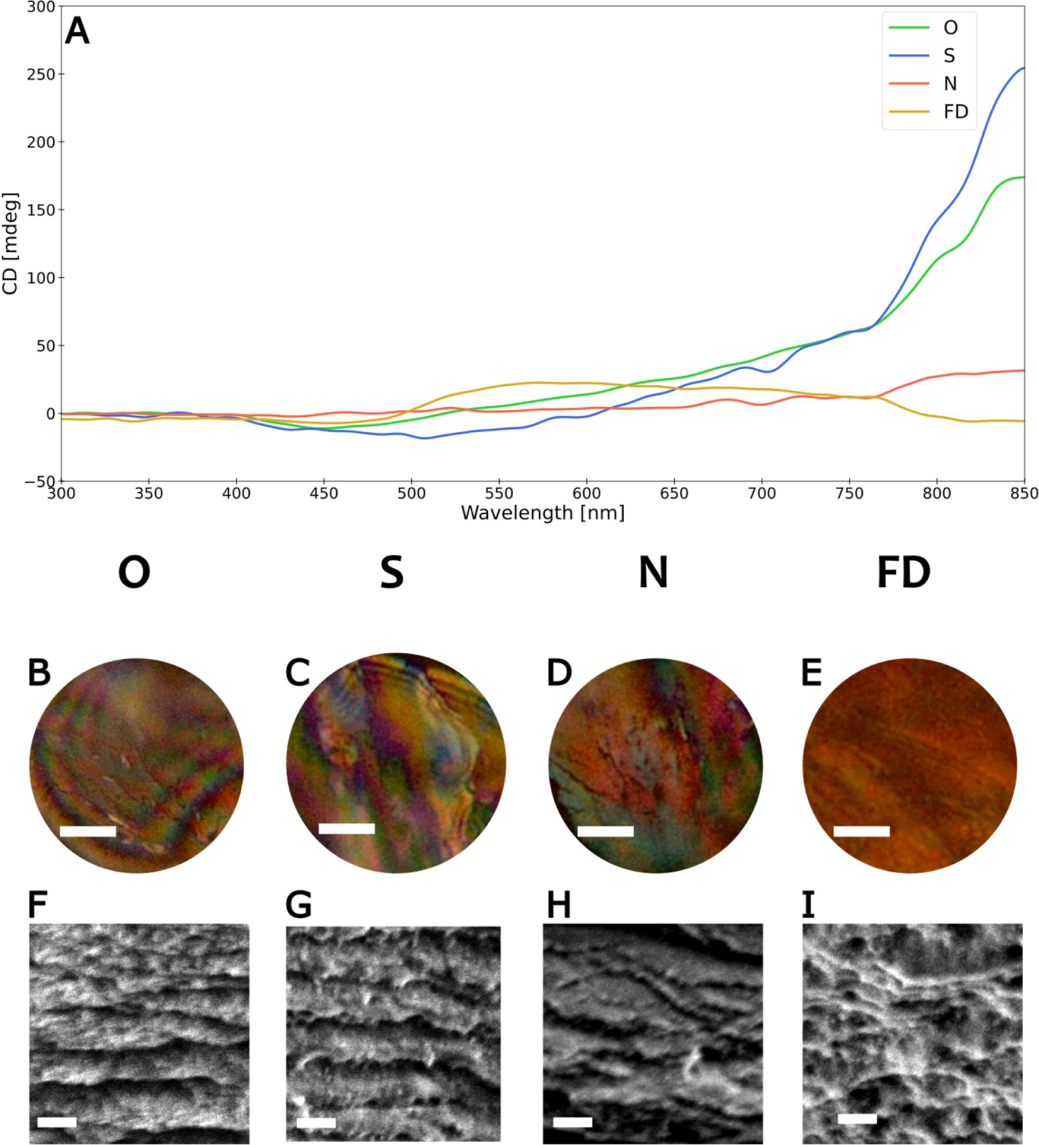
Comparative Characterization of CNC-IONPs-QDs films subjected to different drying protocols. (A) Circular dichroism (CD) spectra across the four film variants: “O” (none), “S” (south), “N” (north), and “FD” (fast dry). (B–E) Polarized optical microscopy (POM) images in reflection mode using left circularly polarized light (LCPL): (B) “O” protocol, (C) “S” protocol, (D) “N” protocol, and (E) “FD” protocol. Scale bars represent 5 μm. (F–I) Scanning electron microscopy (SEM) cross-sectional views: (F) “O” protocol, (G) “S” protocol, (H) “N” protocol, and (I) “FD” protocol. Scale bars are set at 500 nm.

Circular dichroism (CD) analysis (Figure 2a) revealed strong positive CD signals in films from the “S” protocol (slow drying on a magnet’s south pole) with the highest signal around a wavelength of 848 nm and to a slightly lesser extent in films from the “O” protocol (slow drying without a magnet) with the highest signal around a wavelength of 850 nm. Both results are indicative of left-handed chiral nematic order. The slightly stronger signal in “S” suggests a higher degree of ordering, possibly due to magnetic alignment. In contrast, films from the “N” protocol (slow drying on a magnet’s north pole) and the “FD” protocol (rapid drying without a magnet) showed significantly reduced CD signals. The highest CD signal for the “N” protocol was around a wavelength of 812 nm, and for the “FD” protocol, the highest CD signal was around a wavelength of 568 nm. For the “FD” protocol, there was no observed significant signal in the longer wavelengths. This shift in the peak wavelength aligns with previous findings that increasing drying temperatures of CNC leads to a blue-shift of the peak CD signal.^72^ Estimation of the signal-to-noise ratio (SNR) using baseline CD spectra (measured without a sample) yields that, compared with the “O” and “S” samples, which exhibit a relatively high SNR, the “N” and the “FD” samples show a much lower SNR around their peak CD values (Figure S5).

Polarized optical microscopy (POM) images, captured in reflection mode under LCPL, further support these findings (Figure 2b–e). While optical birefringence textures appear in all films, their patterns differ. The “O” and “S” films display a periodic “fingerprint” texture of evenly spaced bright and dark bands, characteristic of a chiral nematic order.^20,21,72^ In contrast, the “N” and “FD” films lack this distinctive pattern, showing only sparse birefringence, further underscoring the absence of the discernible chiral nematic structure in these samples.

Supplementary to CD and POM findings, SEM cross-sectional images (Figure 2f–i) provide consistent evidence for the presence of a chiral nematic order. Films prepared by “O” and “S” protocols show an ordered layered structure that resembles previously reported chiral nematic CNC SEM images.^36,73−75^ Conversely, the “N” and “FD” films exhibit irregular and less defined layered structures, indicating a lower degree of chiral nematic order.

### 2.3 Light Polarization and Electric Field Impact on Spectral Shift

Optical spectra measurements for each device variant (“S”, “O”, “N”, and “FD”) were conducted under RCPL and LCPL illumination, with the incident light direction set at 45° (Figure 1b). Initial measurements without an applied electric field were followed by tests under varying E-field magnitudes to assess their effect on the emission spectra. Between measurements, devices were discharged to neutralize persistent charge separation, thereby revealing a capacitor-like behavior. This separation was initiated by light excitation, even in the absence of an external voltage bias. Without the discharge step, a carryover effect became apparent, whereby at the start of subsequent measurements, the emission already exhibited the red-shift observed at the end of the previous session. This implies the film’s ability to hold voltage and sustain charge separation, even after stopping illumination or the removal of an external electric field.

**Figure 3:**
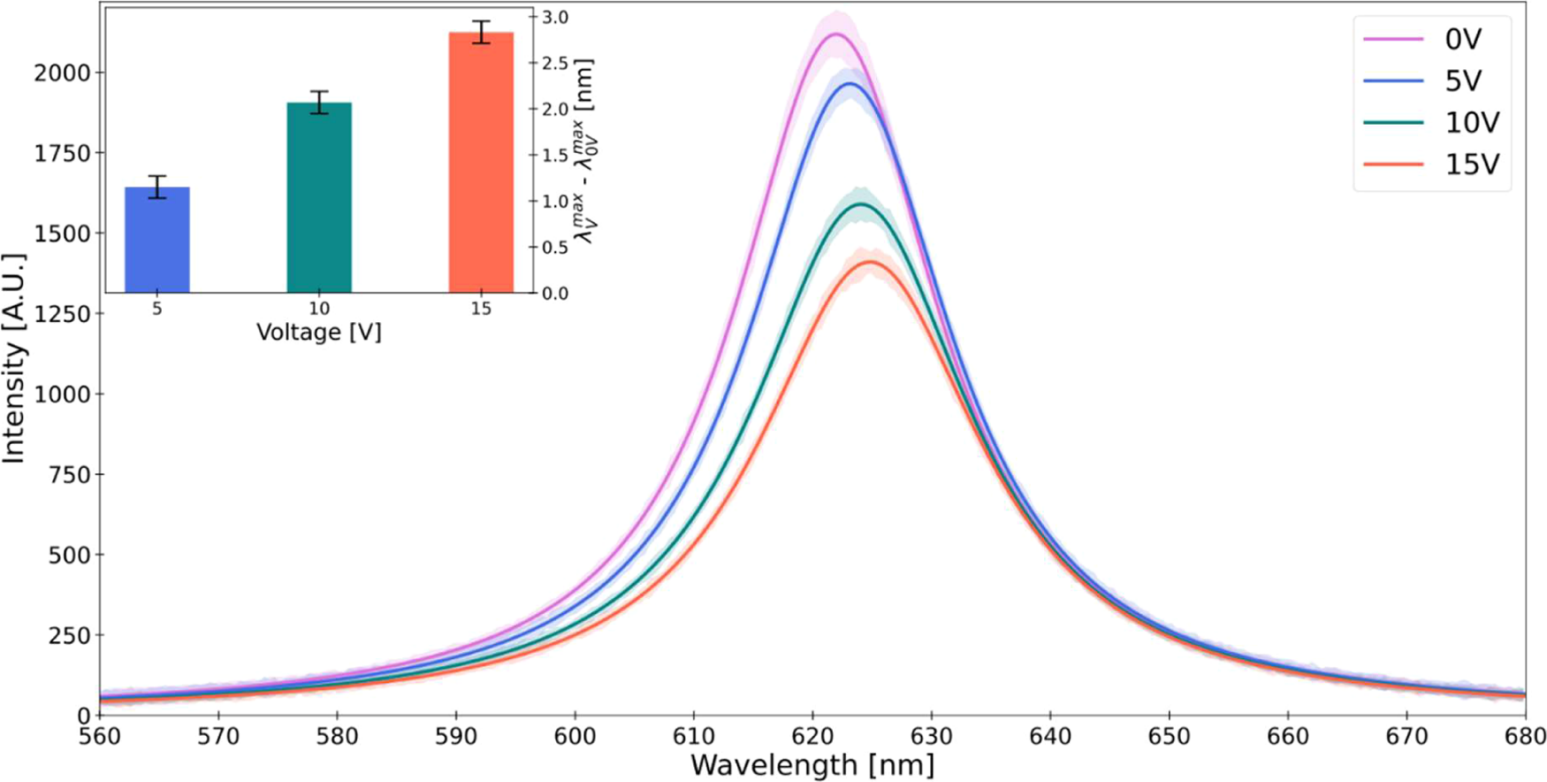
Spectral response to an applied electric field. Spectral shift response under varying E-field magnitudes in a device “O” dried without a magnet and excited with left CPL. Curves represent averaged emission spectra across sessions for each bias level. The shaded regions represent the standard deviation of the emission spectra across the sessions for each bias level. Inset: Corresponding average emission peak shifts were observed per bias level. Error bars represent the standard deviations of the average emission peak shifts.

The average spectral shift responses of the O device under LCPL excitation to various external electric field magnitudes are depicted in Figure 3. For each bias level (0, 5, 10, and 15 V), 20 emission measurement sessions were conducted, with the device being discharged after each. A Lorentzian fitting function was applied to the data from each session, identifying the peak wavelength (λ^max^). A progressive red shift in the average emission spectra of the QDs as the external electric field magnitude increases is shown in Figure 3, with the corresponding shift of λ^max^ shown in the inset. This trend is consistent with prior studies,^54,55,57,76^ but the magnitude of the effect observed in the CNC-IONPs-QDs film is significant, considering the applied electric field magnitudes.

To estimate the electric field magnitude experienced by the CNC-IONPs-QDs film due to the externally applied bias, we used an ideal plate capacitor approximation, treating the ITO, Al_2_O_3_, and CNC-IONPs-QDs layers as three capacitors in series, each with specific dielectric constants and thickness. For instance, a 15 V external bias applied to the whole device resulted in an estimated electric field of about 120 kV/cm on the CNC-IONPs-QDs film, leading to a red shift of 2.83 ± 0.03 nm (9 meV) in the emission peak. Such sizable emission peak shifts, as observed under external electric fields of about 120 kV/cm, are unusual for symmetric spherical QD ensembles with random orientations like ours, where red shifts below 1 nm were measured.^55^ With an electric field magnitude tripled to 390 kV/cm, a 2.3 nm red shift was previously reported.^76^ At an even higher field magnitude of 600 kV/cm, about 5–6 times ours, a ∼6.2 nm shift was documented.^57^

Typically, in a QD ensemble, transient local electric fields from charge carriers on or near QD surfaces exhibit random orientations, often nullifying the average dipole component relative to an external field.^54^ However, the observed significant emission peak shifts suggest the formation of local electric fields around the QDs in our film that have a consistent average orientation with a component that aligns with the externally applied field.

We conjecture that a CISS-driven charge separation is facilitated due to the chiral nematic structure of the CNC-IONPs-QDs film. The inherent asymmetry of the CISS would then lead to a correlated orientation of the resulting local electric fields. To test this, CPL was used to excite the QDs in devices with varying degrees of chiral nematic order. The choice of light polarization (left or right) affects the electron–hole spin states.^43,44^ Consistent with CISS, this should affect the probability of charge transfer through chiral nematic structures, enhancing or reducing the resulting charge separation.^9,14,77^ Given the left-handedness of the chiral nematic CNC, one circular polarization is expected to result in more pronounced and persistent local charge separations around the QDs compared to the other.^78^ Thus, aligned with CISS, differences in spectral shifts should be observable in CNC-IONPs-QDs films with a higher degree of chiral nematic order at a given bias voltage when responses are compared between LCPL and RCPL.

**Figure 4:**
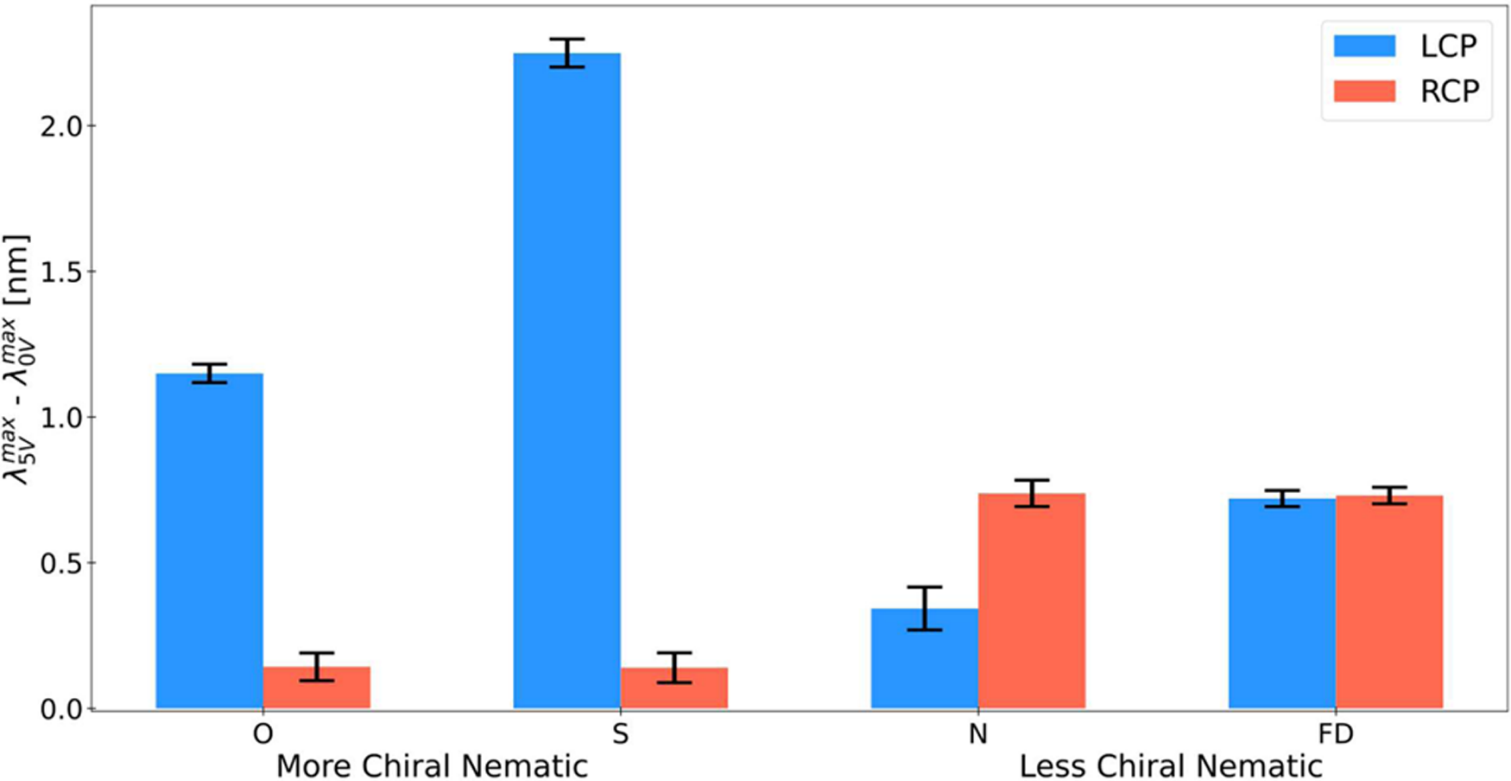
Mean emission peak shift in response to an applied electric field for different light polarizations and chiral orders. Mean emission peak shifts are depicted in response to voltage bias of 5 V applied to each one of the device variants (“O”, “S”, “N”, and “FD”) that were excited with left (L) or right (R) CPL. Error bars represent the standard errors of the mean emission peak shifts and encompass both peak estimation errors within individual measurements (from Lorentzian fits) and peak variations between all measurements. “S” and “N” device variants were dried on south and north magnetic fields, respectively. No external magnetic fields were used during the optical and electrical measurements of any film.

Examining this proposition, the mean shift in the emission peak (λ^max^) under a 5 V external bias (40 kV/cm on the CNC-IONPs-QDs film) is illustrated in Figure 4. This is measured across the four device variants: “O”, “S”, “N”, and “FD”, and excited with either LCPL or RCPL (the state of polarization of the exciting light in Figure S6). As before, 20 emission measurement sessions were conducted for each device variant and light polarization and discharging of the device followed each session, and the peak wavelength (λ^max^) was estimated from the Lorentzian fit. Note that the top and bottom contacts in the device are not the same, thereby generating an intrinsic difference in work functions between the two metals. Error bars represent standard errors of the mean spectral shift, reflecting errors in peak estimations from Lorentzian fits within individual sessions (intrasession) and the variability of peak shifts across sessions (intersession).

Our findings reveal a significant difference in the emission peak shift profile between devices with more chiral nematic order, i.e., “O” and “S”, and devices with less chiral nematic order, i.e., “N” and “FD”. When excited with LCPL, the peak shift under external voltage bias for the more chiral nematic group was notably larger, exhibiting an order of magnitude difference compared with RCPL excitation. In the “O” device, an average 1.15 ± 0.03 nm shift (3.7 meV) was noted for LCPL excitation compared to an average 0.14 ± 0.05 nm shift (0.45 meV) for RCPL. The “S” device further accentuated this difference, with an average 2.25 ± 0.05 nm shift (7.2 meV) for LCPL excitation versus an average 0.14 ± 0.05 nm shift (0.45 meV) for RCPL. However, in the less chiral nematic devices, i.e., “N” and “FD”, this significant difference between LCPL and RCPL excitation was not observed.^28^ Specifically, the “N” device exhibited an average 0.34 ± 0.07 nm shift (1.1 meV) for LCPL excitation against an average 0.74 ± 0.05 nm shift (2.38 meV) for RCPL, while the “FD” device showed an average 0.72 ± 0.03 nm shift (2.32 meV) for LCPL excitation compared to an average 0.73 ± 0.03 nm shift (2.35 meV) for RCPL, suggesting a more uniform response irrespective of the light polarization.

The differential response between LCPL and RCPL is consistent with the chiral nematic structure of CNC-IONPs-QDs facilitating the CISS-driven charge separation. Furthermore, these observations are unlikely to be influenced by differences in the optical properties of CNC-IONPs-QDs as photonic films (differences in selective LCPL reflection and RCPL transmission),^21^ as the differences in CD signals around the 620 nm emission region of the QDs are minimal, e.g., about 0.3 mdeg between “S” and “N” (Figure 2a), this negligible variation extends to the 450 nm exciting laser region as well.

**Figure 5:**
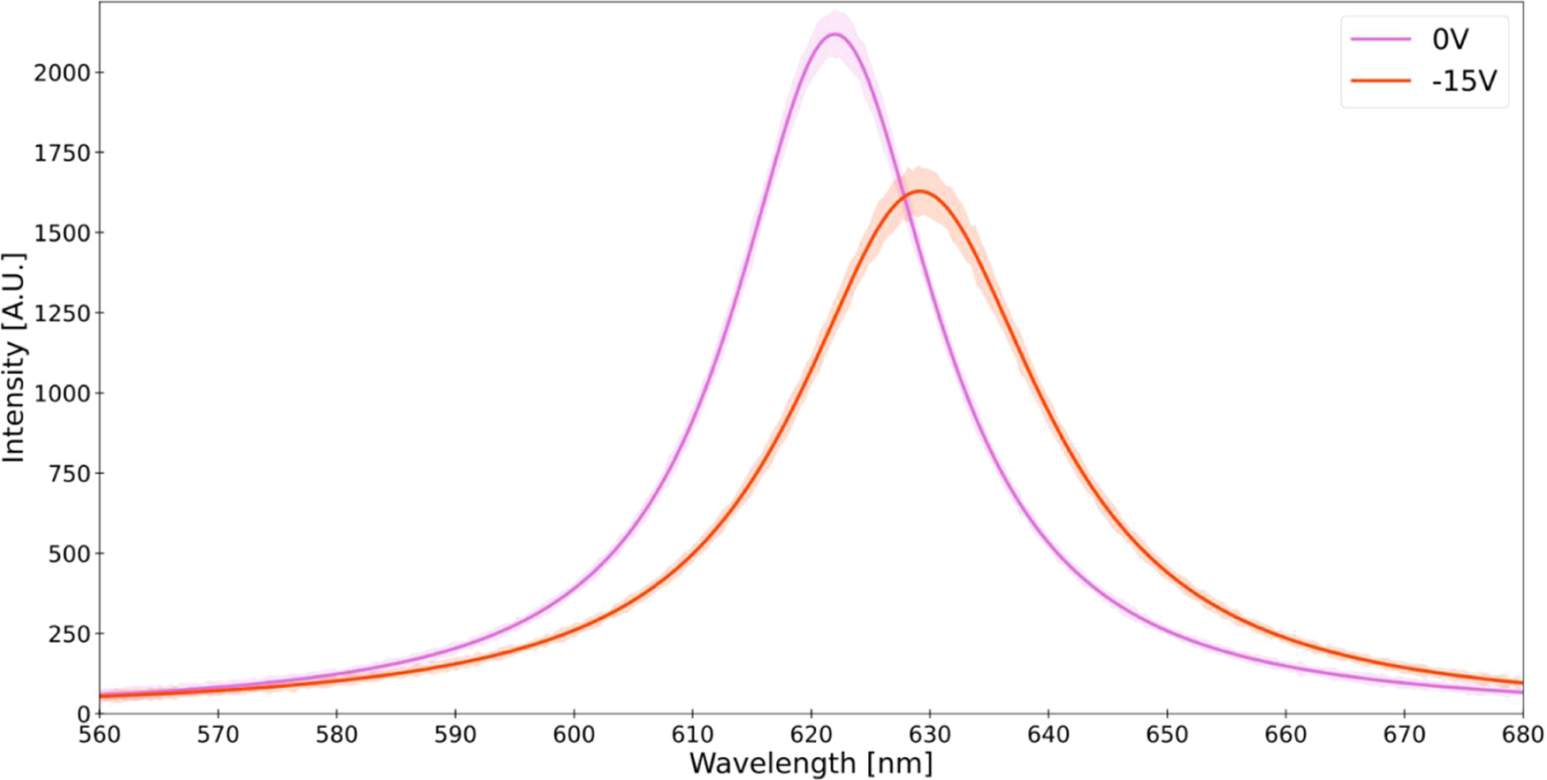
Maximal spectral response to an applied electric field with reversed bias. Spectral shift response under 0 and −15 V (0–120 kV/cm) negative bias (with respect to Figure 3) in a device “O” dried without a magnet and excited with left CP. The figure shows the averaged emission spectra across sessions for each bias level. The shaded regions represent the standard deviation of the emission spectra across sessions for each bias level.

We next examined the effect of reversing the bias voltage polarity on spectral shifts, anticipating differences in response due to CISS. The spectral response under a 15 V negative bias for the chiral nematic “O” device variant, which was excited with LCPL, is shown in Figure 5. This setup mirrors the approach in Figure 3, differing only in the bias direction. Notably, a negative bias induced a significant average red shift of 7.2 ± 0.1 nm (22.8 meV), 2.5 times larger than the 2.8 ± 0.1 nm (9.09 meV) average shift observed under a positive bias under identical conditions (Figure 3). This pronounced asymmetry in shift magnitude under reversed electric fields diverges from previous findings of symmetric spectral shifts within QD ensembles.^76^ The average 7.2 nm shift is the largest we recorded across all tested device configurations, light polarizations, and voltage biases within the 0–15 V range. For comparison, under the same 15 V negative bias, excitation with RCPL induced a smaller average red shift of 2.6 ± 0.1 nm (Figure S7).

### 2.4 Emission Dynamics

Empirical evidence suggests that after charge ejection from excited QDs and subsequent CISS-driven separation, charges may tunnel to trap states within the CNC-IONPs-QDs film, even after spin relaxation.^79^ Alternative models propose that these charges interact with the film’s dielectric and polarizable environment, stabilizing in a self-trapped state due to the reaction field. This stabilization process is significantly influenced by the material’s dielectric properties;^80,81^ notably, the CNC-IONPs-QDs films in this study had a relatively high dielectric permittivity averaging 8 ± 2. This suggests the potential for continuous accumulation of photoexcited charges and the buildup of local, persisting dipoles within the film. Consequently, without actively discharging the device, further spectral shifts with each subsequent light excitation are expected, which can be observed even without an external voltage bias.

**Figure 6:**
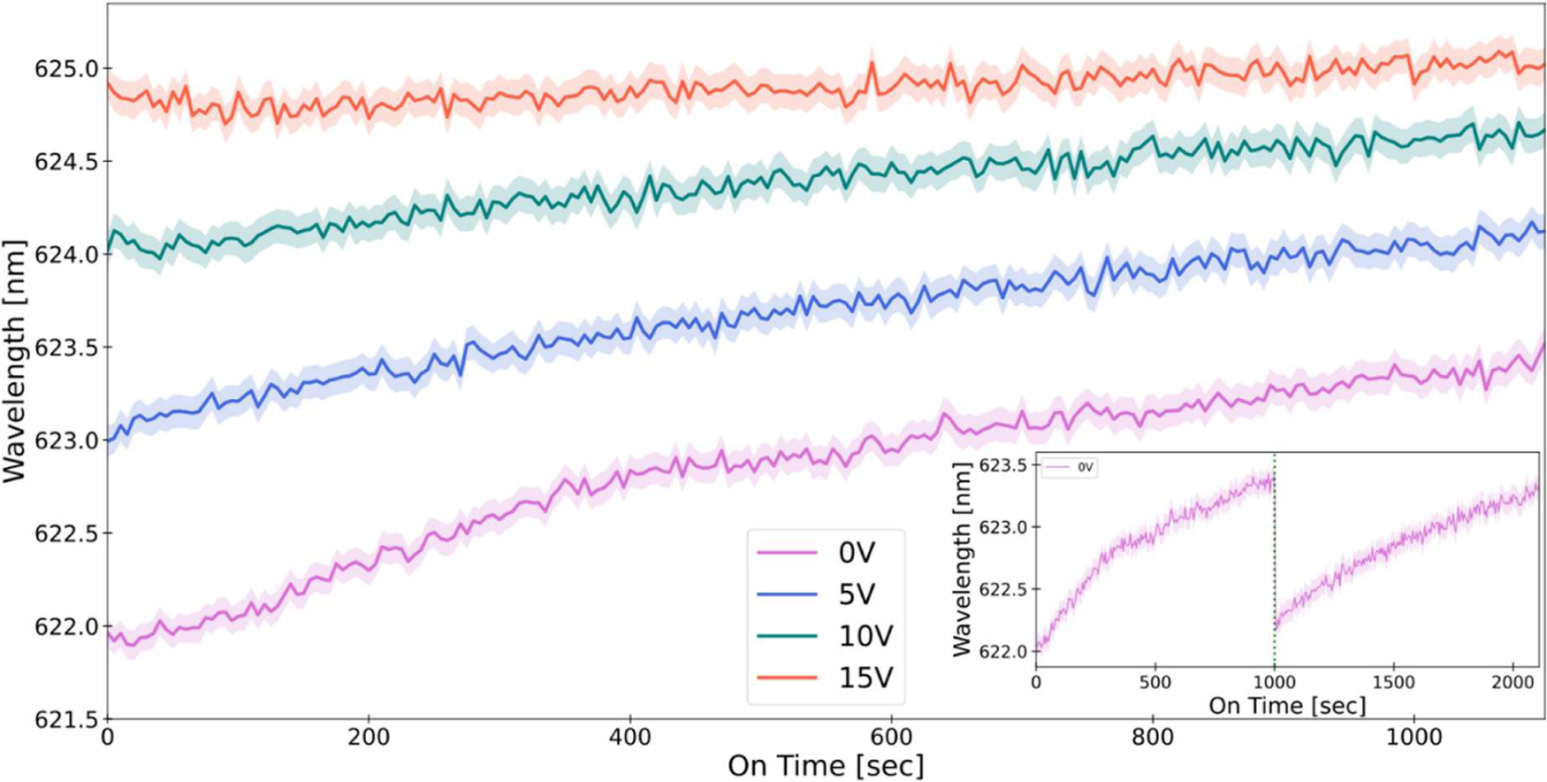
Dynamics of the emission peak wavelength. The figure tracks the evolution of the maximum emission peak wavelength (λ^max^) under different bias levels (0, 5, 10, and 15 V) for device variant “O” dried without a magnet and excited with left CPL. Inset: two-round emission peak wavelength dynamics separated by a discharge, no bias applied. The inset shows two consecutive sets of measurements of the evolution of the maximum emission peak wavelength (λ^max^) for a device variant dried without a magnet and excited with left CPL and with no applied bias (0 V). Following the initial data set acquisition session, the device was left to rest disconnected from the power source for 24 h and then underwent a single discharge (represented by a dotted green line) before the second data acquisition session commenced.

The evolution of the maximum emission peak wavelength (λ^max^) at each applied bias level (0, 5, 10, 15 V), for the chiral nematic device variant “O”, which was subjected to excitation via LCPL, is illustrated in Figure 6. We conducted 220 emission measurement sessions for each bias level. In each recording period, the excitation laser was active for 10 s, recording two sessions, followed by an 11 s interval with the laser off to allow the device to rest and cool. Notably, unlike previous experiments, the device was not discharged after each session. The time series in Figure 6 presents only the recording periods, excluding the nonrecording intervals when the laser was off.

A noticeable upward trend for the 0, 5, and 10 V lines indicates a red shift in the emission peak wavelength with each subsequent light excitation. This effect is particularly evident in the 0 V time series. Furthermore, the initial vertical gap on the *Y*-axis at *t* = 0 between the peak wavelength of each positive bias level and the 0 V level corresponds to the spectral shift measured for each bias level, mirroring the spectral shift shown in Figure 3 for the same device, “O”, and LCPL excitation. Notably, after 220 light excitations (1100 s), the peak wavelength in the 0 V graph reaches a red shift of ∼1.5 nm, similar to that induced by a 5 V bias, as evident from the initial peak (*t* = 0) wavelength of the 5 V graph.

Figure 6 inset illustrates the course of an experiment mirroring the procedures from Figure 6, conducted only under zero bias (0 V), with the device variant O, and excited with LCPL. The experiment comprised two consecutive sets of 220 sessions each, and after the first set, the device was left to rest disconnected from the power source for 24 h and then discharged once by a 20 s short circuit before the second set commenced. An upward trajectory in the emission peak wavelength was observed for each set of measurements, echoing the red shift pattern observed in Figure 6 with each successive light excitation. Importantly, following the discharge of the device (noted at the second 1000 marker), there is a discernible reduction in the emission peak wavelength at the beginning of the second set, indicating a reversion to a value similar to the initial state of the first set. These findings suggest that the cumulative light excitations not only yielded further localized charge separations within the CNC-IONPs-QDs film through metastable spin polarization but also that this process appears to contribute to a substantial and enduring charge separation across the device’s length scales, exceeding 1 μm, and results in a built-up dipole across the device.

## 3 Conclusions

In recent studies, CNC films and CNC-based aerogels have been utilized as integral components in electronic and energy storage devices such as supercapacitors. These applications primarily exploit the chiral nematic structure of CNC as a scaffold, which provides enhanced mechanical properties through its highly organized order, enables homogeneous dispersion of nanoparticles, and promotes an ordered pore network conducive to homogeneous ionic infiltration and transport.^40,82,83^ Our study, however, adopts a distinct approach by showing that the CISS effect, previously demonstrated to facilitate charge separation in chiral molecules, can be similarly applied to the chiral nematic CNC films. We have generated a hybrid material, CNC-IONPs-QDs, enabling the formation of charge carriers with well-defined spin states upon photoexcitation with CPL. Leveraging the spin-filtering capability of its chiral nematic structure via the CISS effect, we have achieved long-lived charge separation in our device, which is explained by a simple phenomenological quantum model (Supporting Information).

In conclusion, we have demonstrated nanoscale, mesoscale, and device-wide microscale CISS-driven charge separation using a device based on CNC—a sustainable, abundant, and low-cost biomaterial, along with semiconductor QDs and magnetic nanoparticles. It shows sensitivity to the circular polarization excitation of the QDs, with significant variations in the QCSE emission peak shifts under an applied electric field that depends on the chiral nematic order. In addition, we report relatively large peak shift magnitudes for an ensemble of spherical QDs with a random orientation. We attribute these results to the CISS effect, enabling efficient room-temperature local charge separation. Moreover, our findings suggest that these local separations promote a durable charge separation across the device’s length scales, extending beyond 1 μm, even in the absence of an external voltage bias.

Leveraging the chiral nematic structure and dielectric properties of CNC and incorporating QDs gives rise to a photovoltaic charge-separating device. This research may enable a diverse array of potential applications, from self-assembled devices that combine photovoltaic cells with electric capacitance to spreadable optical hybrid biosensors.

## 4 Experimental Section

### 4.1 CNC Preparation

CNCs were synthesized from cellulose pulp (Meloda Ltd.) via sulfuric acid hydrolysis,^19,84,85^ yielding a 2 wt % suspension with a pH of 3.2. The resultant rod-like particles were determined by TEM to be between 100–200 nm in length and 5–10 nm in diameter (Figure S1), aligning with previous literature findings.^19,28^

### 4.2 CNC-IONPs Composites Preparation

CNC-IONPs composites have been synthesized by the method proposed by Chen et al.^41^ Forty grams of a 2 wt % suspension of CNC particles was diluted with 160 g of DDW to achieve a 0.4 wt % suspension. The diluted suspension is heated to 70 °C. Then, 0.17 g of ferric chloride hexahydrate and 0.11 g of ferrous sulfate heptahydrate were added under magnetic stirring. A 400 μL portion of ammonium hydroxide was diluted to 20 mL with DDW and preheated to 70 °C. The base solution was added to the CNC suspension and reacted for 15 min; the resultant solution was quickly cooled to room temperature using an ice bath. Centrifugation and dialysis were employed to remove residual reactants and impurities, and the final suspension was concentrated to 1.125 wt % CNC and 0.375 wt % iron oxide. See Figure S2.

### 4.3 CNC-IONPs-QDs Suspension Preparation

Nine hundred microliters of the CNC-IONPs suspension was mixed with 100 μL of 1 mg/mL CdSe/ZnS quantum dots in H_2_O (9.5 ± 0.5 nm total diameter as determined from TEM images, Figure S3, 620 nm emission peak, MKnano). The resultant CNC-IONPs-QDs suspension was ultrasonicated using a Hielscher sonicator (UP200Ht, 200 W; 26 kHz) with a 1 s on–1 s off frequency for 60 min at 20% amplitude.

### 4.4 Device Fabrication

All fabrication processes were conducted in a cleanroom facility. A silicon/silicon dioxide (Si/SiO_2_) wafer, measuring 6 mm by 6 mm with a thickness of 0.3 mm, was coated on one side, with sequential deposition of 15 nm titanium (Ti) and 150 nm gold (Au) by evaporation. This was followed by a sequential acetone boil, sonication in acetone and isopropanol, and ethanol rinse, succeeded by ozone ashing and 20 min of ethanol incubation to ensure a clean Au surface. To enhance the film’s adhesion, 40 μL of a 1% aqueous solution of branched polyethylenimine (PEI) (*M*_W_ = 25 000 g/mol, *M*_n_ = 10 000 g/mol, Merck/Sigma-Aldrich) was deposited onto the Au surface, left for 30 min, and then gently washed using DDW. A protective Kapton tape was applied in the corner of the Au surface to ensure a region for electrical contact before film deposition. The CNC-IONPs-QDs suspension (40 μL) was then gently drop-cast onto the Au surface and left to evaporate. Four film variants were prepared: “S” positioned on the south pole of a magnet and slow-dried, “N” positioned on the north pole of a magnet and slow-dried, “O” slow-dried without a magnet, or “FD” fast dried without a magnet. The magnet condition entailed a constant magnetic field of 200 mT generated by a disk-like magnet beneath a Petri dish. Slow-dried conditions entailed 96 h at 45% relative humidity (RH) and 15 °C until constant weight of the film was achieved, while fast-dry conditions entailed 80 °C for 4 h in an oven. Postdrying, a 30 nm Al_2_O_3_ insulation layer was deposited via atomic layer deposition (ALD) on top of the dried CNC-IONPs-QDs film. A 100 nm layer of transparent indium tin oxide (ITO) (3 mm × 3 mm) was then sputtered in the middle through a shadow mask. Subsequently, a second shadow mask was employed, upon which a dual layer of 15 nm chromium (Cr) and 150 nm Au was evaporated atop the ITO, serving as a contact. Thickness measurements of the CNC-IONPs-QDs dry film were performed using a DektakXT stylus profilometer and a Contour GT Optical Profilometer from Bruker.

### 4.5 Electrical Measurements

A Keysight B2900A Precision Source/Measure Unit was used for a two-probe measurement scheme. Voltage bias (0, 5, 10, and 15 V).

### 4.6 Optical Setup

A 450 nm, 4.5 mW laser (Thorlabs, CPS4509), modulated by a shutter actuated by a Nema-17 stepper motor, was controlled by Arduino Uno. Circular polarization is achieved by passing a continuous-wave laser beam through an achromatic 1/4 waveplate (Thorlabs, 400–800 nm, AQWP05M-600). Focusing on the sample was achieved by a convex lens with a focal length of 50 mm (Thorlabs, LA1131). The emitted fluorescence is collected using a microscope objective, 5× magnification, 0.12 NA (Zeiss, 440120 Achrostigmat). It then passes through a high-pass filter with 550 nm cut-on wavelength (Thorlabs, FELH0550), focused using another convex lens as detailed above, and is coupled to an optical fiber that is connected to a spectrometer (Ocean Optics, Flame Miniature Spectrometer, FLAME-S-XR1-ES). The state of polarization is measured and aligned prior to the experiment using a PAX1000VIS Polarimeter (Thorlabs) with the PAX1000 series polarimeter software package, version 1.4.

### 4.7 Cryo-TEM (Cryogenic Transmission Electron Microscopy)

Direct imaging of the CNCs in aqueous solution was performed by Cryo-TEM. Samples were prepared by applying a 2.5 μL drop of 1% CNC solution to a glow discharge TEM grid (300 mesh Cu Lacey substrate, Ted Pella, Ltd.). The excess liquid was blotted, and the specimen was vitrified by rapid plunging into liquid ethane precooled by liquid nitrogen using Vitrobot (Mark IV, FEI). The vitrified samples were examined at −179 °C using an FEI Tecnai 12 G^2^ TWIN TEM operated at 120 kV and equipped with a Gatan model 626 cold stage. The images were recorded in low dose mode using a 4K × 4K FEI Eagle CCD camera.

### 4.8 Negative Staining TEM

A 2.5 μL drop of 0.1% CNCs was applied to a glow-discharged TEM grid (carbon support film on 300 mesh Cu grids, Ted Pella, Ltd.) for 30 s. The excess liquid was then blotted with filter paper, and the grids were incubated with a 2% uranyl acetate stain for 30 s following blotting. They were then allowed to dry in air for few hours before they were studied by an FEI Tecnai 12 G^2^ TWIN TEM operated at 120 kV.

### 4.9 TEM

2.5 μL drop of QD solution (0.2 mg/mL) was applied to a glow-discharged TEM grid (carbon support film on 300 mesh Cu grids, Ted Pella, Ltd.) for 10 s. The excess liquid was then blotted with filter paper, and the grids were allowed to dry in air for few hours. The sample was then studied by an FEI Tecnai 12 G^2^ TWIN TEM operated at 120 kV.

### 4.10 STEM-in-TEM

STEM and energy-dispersive X-ray spectroscopy (EDS) analyses were conducted using an Aberration Probe-Corrected S/TEM Themis Z G3 (Thermo Fisher Scientific) operated at 300 kV and was equipped with a high-angle annular dark field detector (Fischione Instruments) and a Super-X EDS detection system (Thermo Fisher Scientific).

### 4.11 SEM

The SEM cross sections of fractured films were observed by an Apreo 2S LoVac (FEI company, a part of Thermo Fisher Scientific) SEM, equipped with a built-in STEM detector and with an UltraDry EDS detector (Thermo Fisher Scientific). The films were fractured after freezing in liquid nitrogen or alternatively embedded in epoxy resin (Epon) and cut using a microtome equipped with a diamond blade. Samples were placed on 1 cm × 1 cm silicon substrates, which were then mounted in aluminum stubs. The acquisition was performed by using an accelerating voltage of 2 or 5 kV.

The samples of the CNC-IONPs suspension for STEM were prepared as follows: a 3 μL drop of 0.2% (or 0.04%) CNC-IONPs suspension was placed on glow-discharged carbon coated 300 mesh copper TEM grids (Ted Pella, Inc.). After about 10 s, the excess liquid was blotted, and the grids were allowed to dry in air.

### 4.12 X-ray Powder Diffraction (XRD)

X-ray powder diffraction measurements were performed on a D8 Advance diffractometer with a LYNXEYE-XE-T detector (Bruker AXS, Karlsruhe, Germany) operating in 1D mode. Low-background quartz sample holders were carefully filled with the powder samples. XRD patterns within the range 2–80° 2θ were recorded at room temperature using Cu Kα radiation (λ = 1.5418 Å) with the following measurement conditions: tube voltage of 40 kV, tube current of 40 mA, step-scan mode with a step size of 0.02° 2θ, and counting time of 0.5 s/step.

The unit-cell parameters and the crystal structure were refined (Rietveld Quantitative Analysis) using TOPAS, Bruker software.

### 4.13 Dielectric Permittivity

The dielectric permittivity of the films was determined using a parallel plate capacitor method.^67^ Capacitance measurements were conducted with a Keithley Instruments DMM6500 6 1/2-digit multimeter (Cleveland, OH). For this, a custom mount designed to hold two 10 × 10 mm^2^ copper (Cu) plates parallel to each other was fabricated through 3D printing, incorporating slits for the Cu plates and clips for electrical contact. The relative permittivity, ε_r_, was calculated from the measured capacitance using the formula ε_r_ = *Cd*/(ε_0_*A*), where *C* is the capacitance in Farads, ε_0_ (taken as 8.854 × 10^–12^) represents the dielectric permittivity of vacuum, *d* is the distance between the Cu electrodes in m, and *A* is the electrode surface area in m^2^. For capacitance measurements on freestanding films, 2 mL of either CNC or CNC-IONPs-QDs suspension was drop-cast into polystyrene Petri dishes and dried according to the four distinct protocols: “O”, “S”, “N“, and “FD”. Subsequently, the dried films were carefully detached from the Petri dish surfaces. The resulting films had an average thickness of 22.25 ± 5.8 μm (measured with a Mitutoyo 543-792 Absolute Digimatic Indicator). The average dielectric permittivity for each film type, pure CNC and CNC-IONPs-QDs, was derived from four distinct films per type, with each film undergoing three separate measurements. After each measurement, the film was removed from the setup and then repositioned for subsequent measurement.

### 4.14 Circular Dichroism (CD)

The CD spectra were recorded on a JASCO J-1500 CD spectrometer (JASCO Corporation, Japan), using continuous scanning, scanning speed: 20 nm/min, spectral bandwidth: 1 nm, and digital integration time: 4 s. For the CD spectra measurements, a selection of films formed from each protocol were gently peeled off the gold substrate just after drying.

### 4.15 Polarized Optical Microscopy (POM)

Optical images of the dried films were obtained in reflection mode, with a left circular polarization configuration, by using an Olympus BX53 M microscope coupled with an Olympus SC50 CCD camera. The images were obtained with 20× and 50× objectives (Olympus, MPlanFL N).

### 4.16 Surface Potentials (ζ-potential)

The ζ-potential was measured by analyzing 2 mL of the suspensions using the Zetasizer instrument (Malvern Instruments Ltd., GB). Before ζ-potential measurements, all samples were sonicated for 4 min at 220 W with pulses of 1 s on and 1 s off. The ζ-potential was obtained from the electrophoretic mobility using the Henry equation with Smoluchowski approximation.^66,86^

### 4.17 Measure the Spectral Response to an Applied Electric Field

Twenty sessions of QD emission measurements for each bias level (0, 5, 10, 15 V); within each session, five consecutive spectra (1 s integration time each) were averaged, followed by device discharge, achieved by short-circuiting for 20 s. Lorentzian functions were fitted to the spectra to identify the peak wavelength (λ^max^).

### 4.18 Measure the Dynamics of the Emission Peak Wavelength

The data presented comprise 220 measurement sessions across each bias level. Every session is part of a cycle, which consists of a “laser on” period of 10 s, immediately followed by an 11 s “laser off” rest interval. During each “laser on” period, two separate 5 s spectra recording sessions occur. Each of these recording sessions involves capturing five consecutive spectra (with an integration time of 1 s each), which are then averaged to produce a single session spectrum. A total of 110 such “laser on” cycles were executed. The device was not discharged at any point during the experiment. The shaded regions represent the standard deviation of the mean emission peak for each period and bias level.

## Supporting Information Available

TEM images and size distribution analysis of CNCs; STEM, EDS, and XRD analysis of CNC-IONPs composites, along with size distribution of IONPs; TEM images and size distribution analysis of CdSe/ZnS QDs; SEM cross-section images and EDS analysis of CNC-IONPs-QDs films; circular dichroism spectra with signal-to-noise ratio estimation; the state of circular polarization of the CPL used for excitation; and spectral response to applied electric fields under negative bias conditions for RCPL excitation. (PDF)
