# Supplementary material for: Chiral Nematic Cellulose Nanocrystal Films for Enhanced Charge Separation and Quantum-Confined Stark Effect
Source: ACS Nano. 2024 Oct 9;18(42):28609–21. doi: 10.1021/acsnano.4c04727 (PMC11503774; doi:10.1021/acsnano.4c04727)
Supplement: Supplementary file 1 — nn4c04727_si_001.pdf [file nn4c04727_si_001.pdf]

# Supporting Information

## Chiral Nematic Cellulose Nanocrystal Films for Enhanced Charge Separation and Quantum-Confined Stark Effect

Gur Aminadav 1,2,3,†, Omer Shoseyov 1,3,†, Shylee Belsey 2, Daniel Voignac 2,3, Shira Yochelis 1,3, Yael Levi-Kalisman 3, Binghai Yan 4, Oded Shoseyov 2,3,* and Yossi Paltiel 1,3,*

1 Department of Applied Physics, The Hebrew University of Jerusalem, Jerusalem 9190401, Israel

2 Department of Plant Sciences and Genetics in Agriculture, Robert H. Smith Faculty of Agriculture, Food and Environment, The Hebrew University of Jerusalem, Rehovot 7612001, Israel

3 The Center for Nanoscience and Nanotechnology, The Hebrew University of Jerusalem Jerusalem 9190401, Israel

4 Department of Condensed Matter Physics, Weizmann Institute of Science, Rehovot 7610001, Israel

***Corresponding Authors**

Yossi Paltiel
Email: paltiel@mail.huji.ac.il

Oded Shoseyov
Email: shoseyov@agri.huji.ac.il

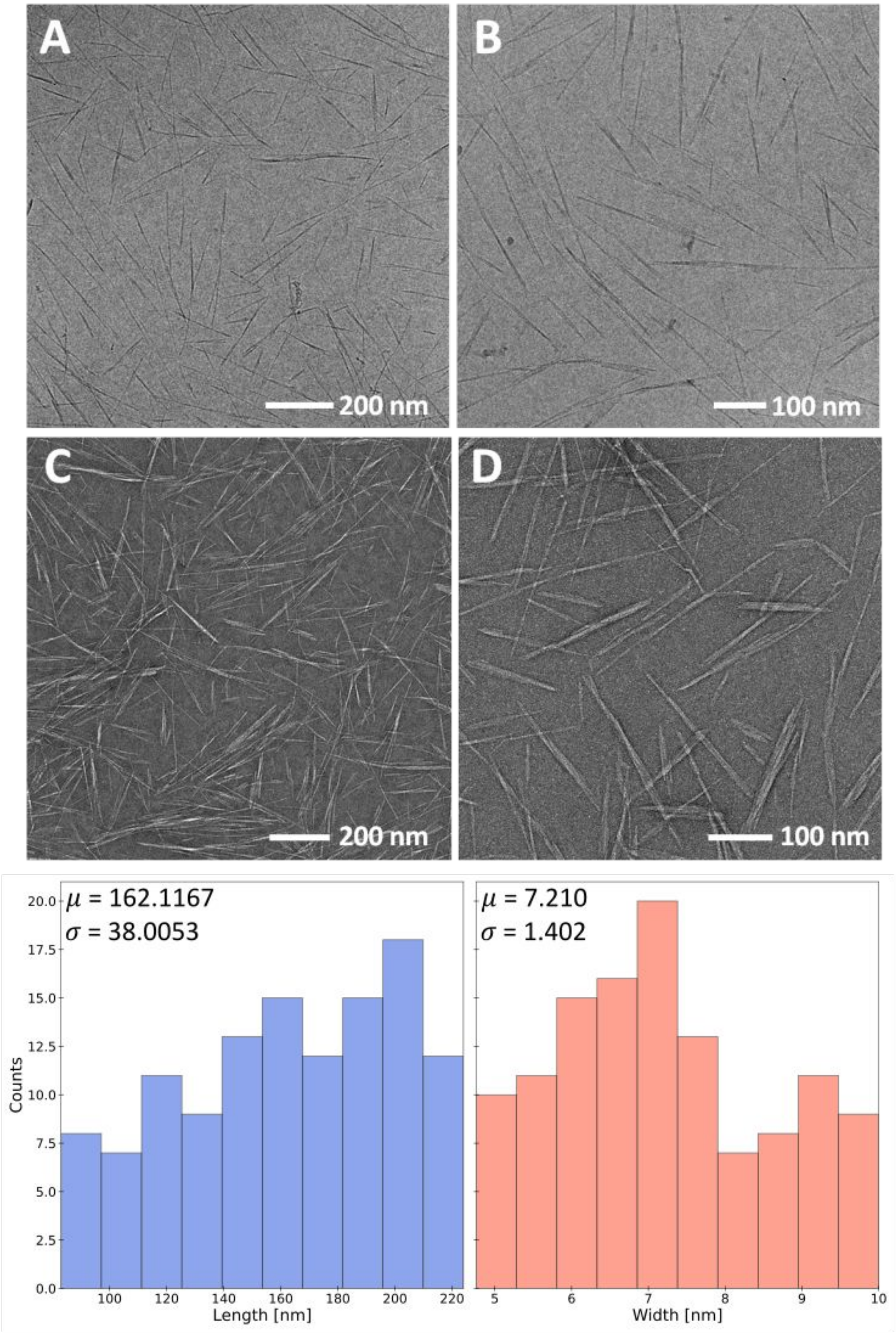


**Figure S1.** Cryo-TEM (A, B) and negative staining TEM (C, D) images of CNCs at two different magnifications. Bottom: Distribution of the length (left) and the width (right) of randomly picked 120 CNC particles measured based on the TEM images.

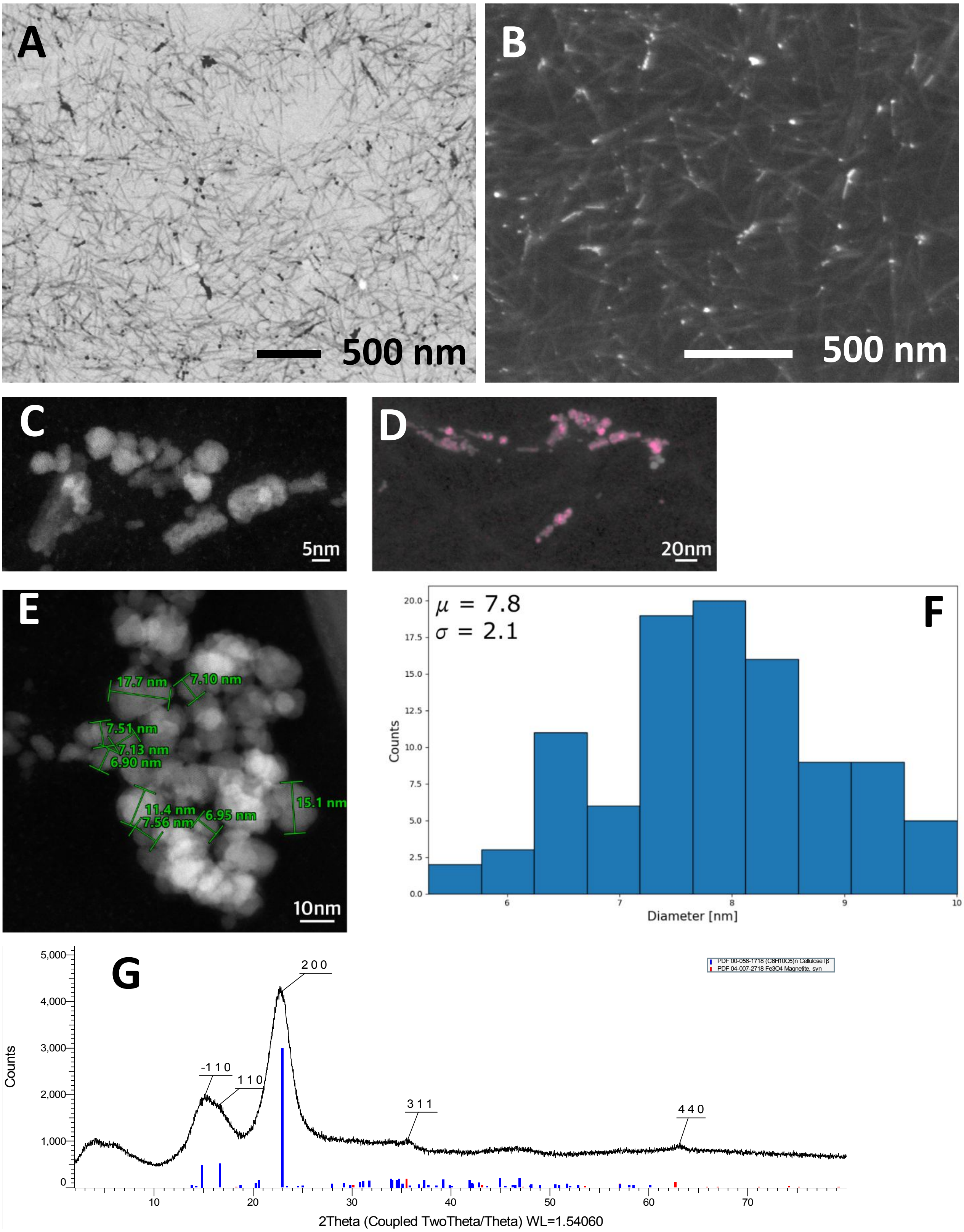


**Figure S2.** Characterization of the CNC-IONPs composites. STEM-in-SEM images of CNC-IONPs composites taken in: Bright field (A), and High-angle annular dark-field (HAADF) mode (B). STEM-in-TEM images of CNC-IONPs composites taken in HAADF mode (C, E). STEM-in-TEM taken in HAADF mode and Energy-Dispersive X-ray Spectroscopy (EDS) in

STEM showing the Fe atoms in pink (D). Distribution of the diameter of randomly picked 100 iron oxide particles measured based on the STEM-in-TEM images (F). X-ray powder diffraction (XRD) measurements with Miller indices for cellulose nanocrystal ordering and iron oxide nanoparticles (G)

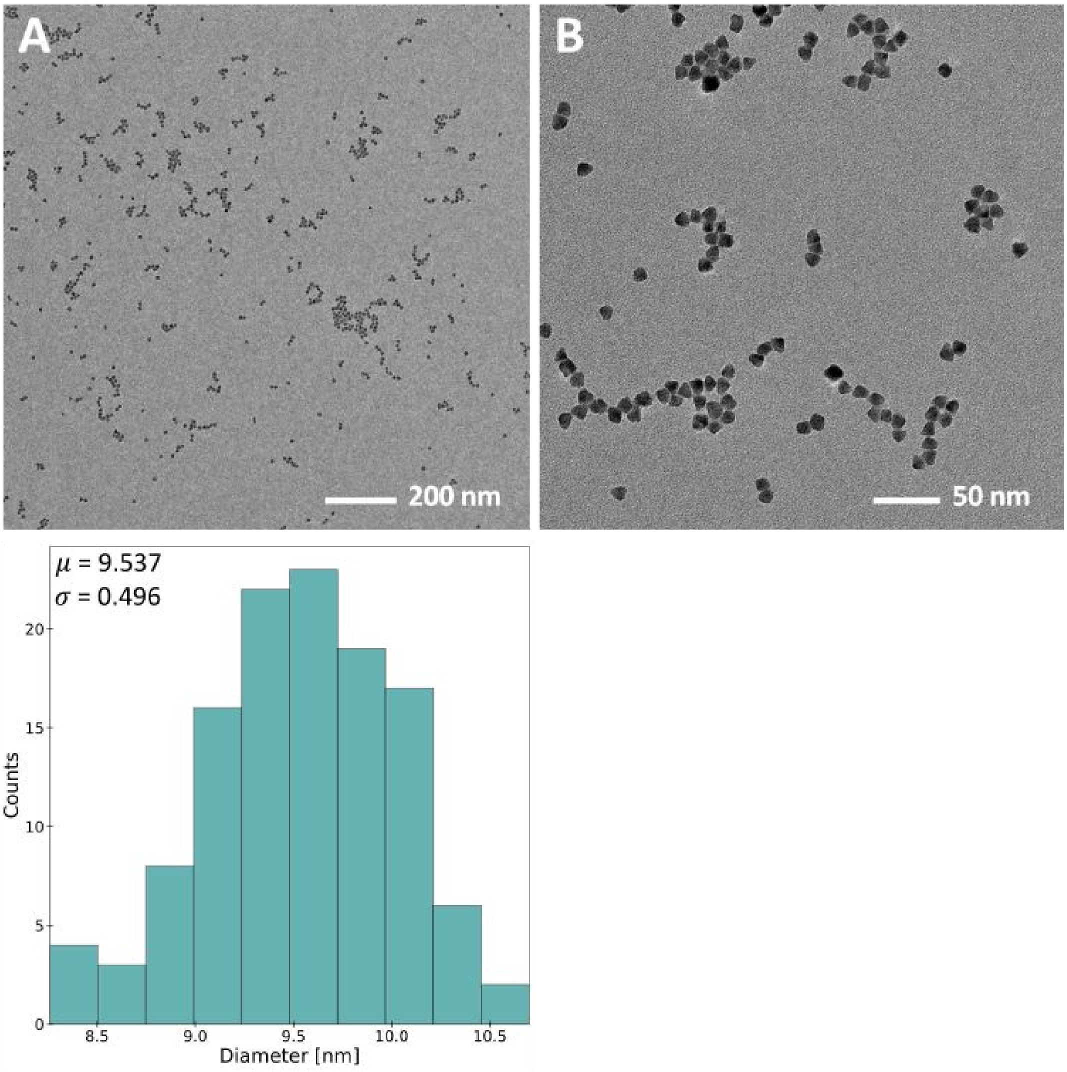


**Figure S3.** TEM images of CdSe/ZnS QDs at two different magnifications. Bottom: Size distribution of the diameter of randomly picked 120 QDs measured based on the TEM images.

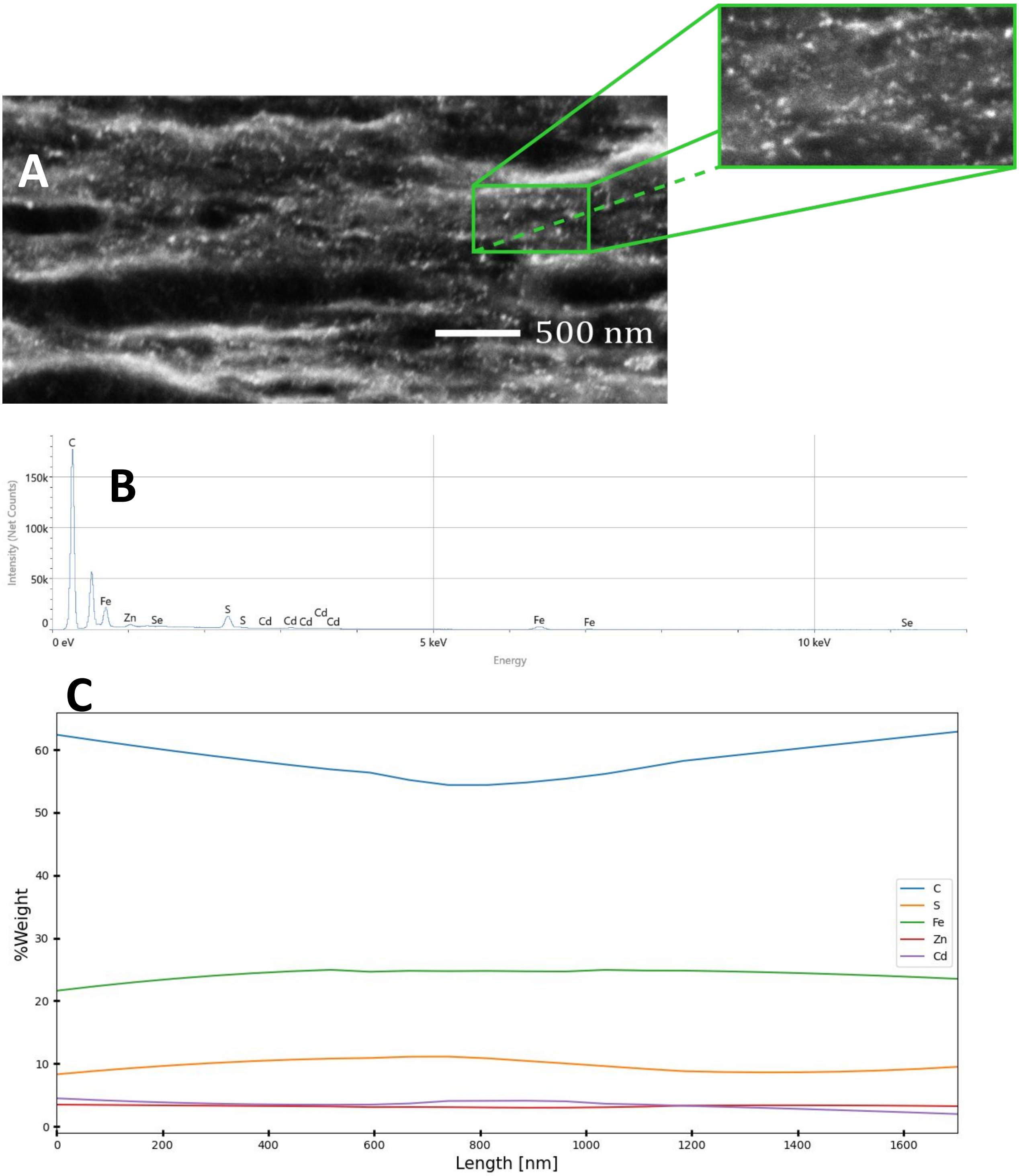


**Figure S4.** Characterization of the dry CNC-IONPs-QDs film. SEM image of CNC-IONPs-QDs film's cross section (microtome cut) of type 'O' (dried slowly without a magnet), showing the dispersion of the IONPs and of the QDs, both appearing as white particles in the CNC matrix (A). On the right: inset showing a zoom in to the area in green. EDS analysis in SEM (B). EDS analysis in SEM along a 1.7 μm line running diagonally to the cross section of the

film, sampling 24 evenly spaced points, showing the weight percent of chosen elements: C, S, Fe, Zn, Cd (C).

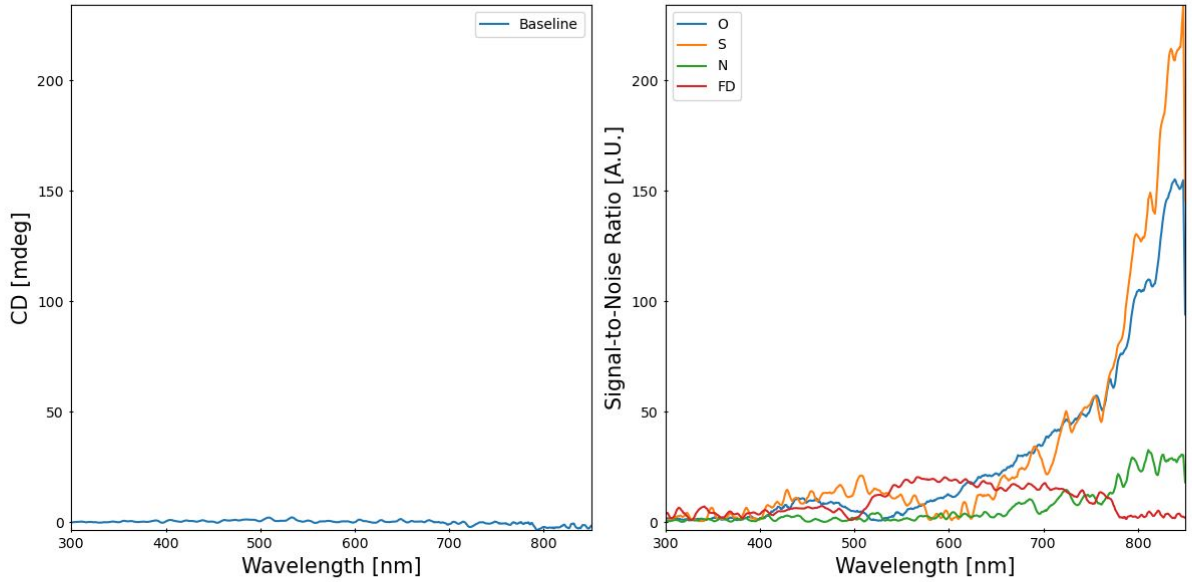


**Figure S5.** Signal-to-noise ratio (SNR) estimation of Circular Dichroism (CD) spectra for the four film variants: 'O' (none), 'S' (south), 'N' (north), and 'FD' (fast dry). Baseline (Left) and SNR (Right). For each film, the SNR was calculated as the absolute value of the difference between the film CD spectrum and a baseline (CD spectra measured without a sample), divided by the standard deviation of the baseline.

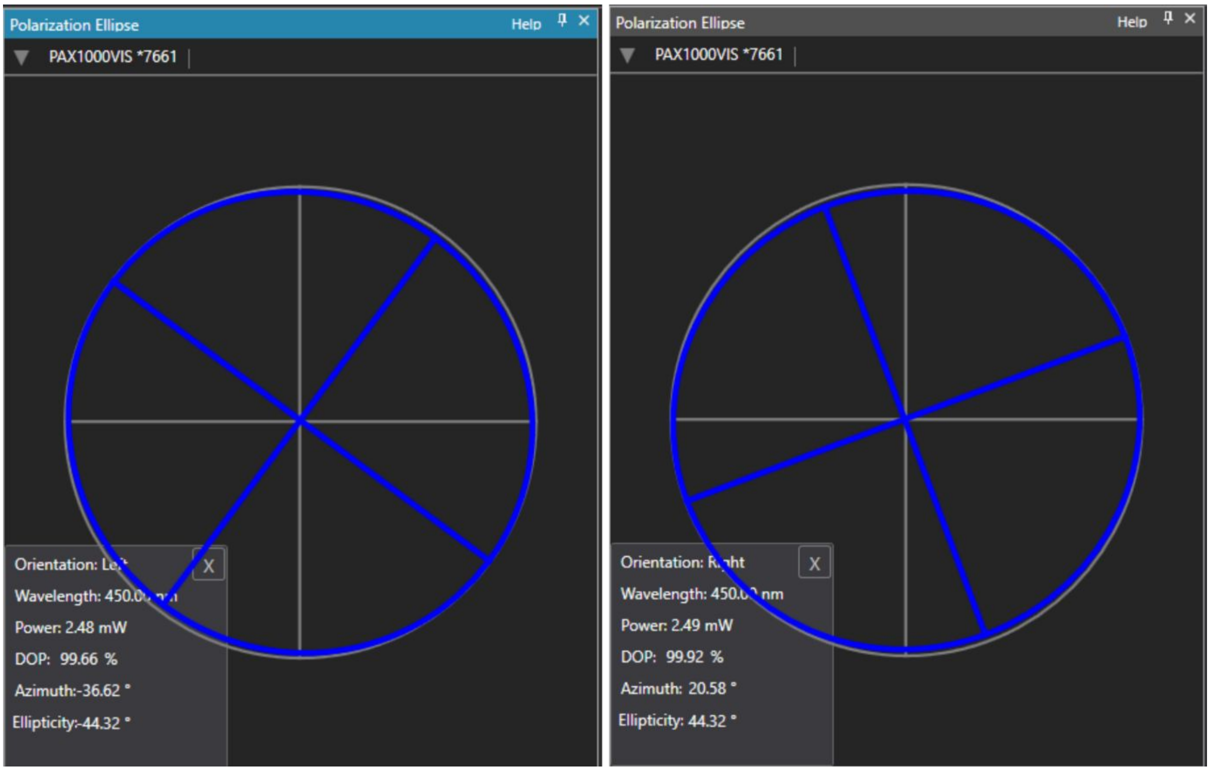


**Figure S6.** The state of polarization of the CPL exciting the sample. A 450 nm, 4.5 mW laser was used for excitation. RCPL (right) and LCPL (left). Circular polarization is achieved by passing a continuous-wave laser beam through an achromatic ¼ waveplate. Focusing on the sample was achieved by a convex lens, with a focal length of 50 mm. The state of polarization is measured using a PAX1000VIS Polarimeter (Thorlabs) with the PAX1000 series polarimeters software package.

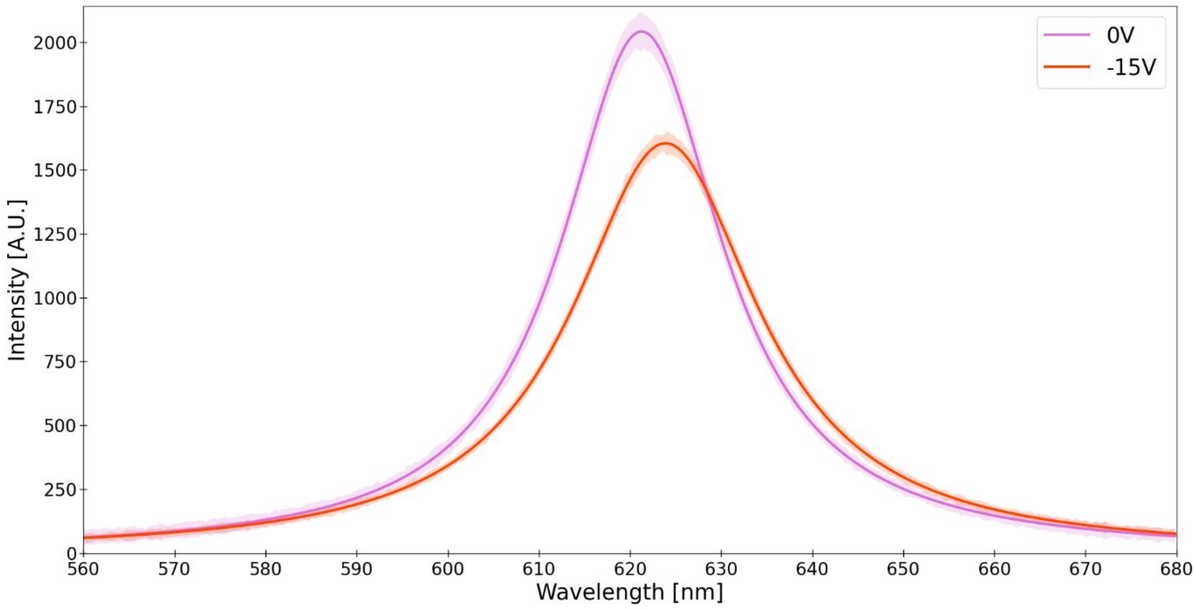


**Figure S7.** Spectral response to an applied electric field, reversed bias. Spectral shift response under 0V and -15V (0 to 120 KV/cm) negative bias (with respect to Fig 3) in a device 'O' dried without magnet and excited with right CPL. The figure compiles 20 sessions of QD emission measurements per each bias level (0V, -15V); within each session, five consecutive spectra (1 sec integration time each) were averaged, followed by device discharge. Lorentzian functions were fitted to the spectra to identify the peak wavelength ($\lambda^{max}$). The figure shows the averaged emission spectra across sessions for each bias level.

## CPL and Induced Polarization

To account for the above observations, the following model is proposed. First, consider another case where the solid, e,g., CdSe, is assumed to be noncentrosymmetric. When shining CPL on a noncentrosymmetric CdSe, electron and hole pairs are excited and lead to a dc photocurrent ($J_{ph}$). We note that quantum states at opposite momentum (± k) carry opposite angular momentum (including both orbital and spin)[1] and opposite velocity when inversion symmetry is broken and the CPL photon also carries angular momentum ($\hbar$). Because of the angular momentum selection rule, RCPL excites states mainly in one momentum valley while LCPL excites states mainly in the opposite valley, leading to excited carriers with opposite velocities. Therefore, RCPL and LCPL generate opposite photocurrent, called injection current or circular photogalvanic effect (CPGE) in literature.[2–4] For the chiral material with random orientation,

which is the case of CdSe nanoparticles in a chiral matrix, the photocurrent direction is along the light propagation axis and current along other directions are averaged to zero. In an open-circuit device, the photocurrent leads to a finite polarization, i.e., an open circuit voltage, $V_{oc} = J_{ph} \cdot R$ , where R is internal resistance. Because R is large in the device, it is not surprising to realize a large voltage. In contrast, $V_{oc}$ of a p-n junction solar cell is limited by the band gap of material. In the injection current scenario, $V_{oc}$ is free from such a constrain and generates a giant electric in the capacitor setup. For the given CPL excitation, the material chirality is the key to determine the photocurrent direction and thus the induced E-field direction.

In the present experiment, we observe a dc current (probed by the E-field in QCSE) generated by irradiating CPL where the CPL handedness controls the dc current direction. This is an inverse process of the anomalous CPL emission reported in chiral polymers,[5] in which the dc current direction controls the CPL handedness. Therefore, it is natural that both phenomena originate in the unique angular momentum[1] in the chirality-light interaction.

## References


(1) Liu, Y.; Xiao, J.; Koo, J.; Yan, B. Chirality-Driven Topological Electronic Structure of DNA-like Materials. *Nature materials* **2021**, *20*, 638–644.

(2) Sipe, J. E.; Shkrebtii, A. I. Second-Order Optical Response in Semiconductors. *Physical Review B* **2000**, *61*, 5337.

(3) De Juan, F.; Grushin, A. G.; Morimoto, T.; Moore, J. E. Quantized Circular Photogalvanic Effect in Weyl Semimetals. *Nature communications* **2017**, *8*, 15995.

(4) Holder, T.; Kaplan, D.; Yan, B. Consequences of Time-Reversal-Symmetry Breaking in the Light-Matter Interaction: Berry Curvature, Quantum Metric, and Diabatic Motion. *Physical Review Research* **2020**, *2*, 033100.

(5) Wan, L.; Liu, Y.; Fuchter, M. J.; Yan, B. Anomalous Circularly Polarized Light Emission in Organic Light-Emitting Diodes Caused by Orbital–Momentum Locking. *Nature Photonics* **2023**, *17*, 193–199.
